# Next‐Generation Sequencing to Identify Genetic Causes of Chinese Patients Affected With Non‐retinal Developmental Eye Disorders

**DOI:** 10.1155/humu/4035027

**Published:** 2026-10-16

**Authors:** Xiaozhen Liu, Ruixuan Jia, Yaning Zhao, Shicheng Yu, Boling Xu, Liping Yang, Jing Hong

**Affiliations:** ^1^ Department of Ophthalmology Peking University Third Hospital Beijing China puh3.net.cn

**Keywords:** *de novo* variants, developmental eye disorders, hereditary eye disease enrichment panel, Rubinstein–Taybi syndrome, whole exome sequencing

## Abstract

To investigate the genetic causes of non‐retinal developmental eye disorders in 14 Chinese different families, we subjected participants to a specific hereditary eye disease enrichment panel (HEDEP) or whole exome sequencing. We further validated disease‐causing variants and performed co‐segregation analyses on the available family members by Sanger sequencing. We classified sequence changes according to the American College of Medical Genetics and Genomics and the Association for Molecular Pathology variant interpretation guidelines. Fourteen probands with diverse clinical diagnoses were analyzed, including microphthalmia (with or without microcornea and congenital cataract or congenital corneal opacification (CCO), four cases), anterior segment dysgenesis (ASD, six cases)—including Peter′s anomaly (PA, one case), PA and microphthalmia (one case), iridocoloboma or aniridia (with microcornea and/or congenital cataract, two cases), and primary congenital glaucoma (PCG, two cases)—congenital cataract (with CCO or microcornea, three cases), and Rubinstein–Taybi syndrome (RSTS, one case). Initial inheritance patterns for 1, 5, and 8 cases were autosomal recessive, autosomal dominant, and sporadic, respectively. We identified 17 (likely) pathogenic variants and one variant of uncertain significance (VUS) in 16 genes, including *BMP4*, *BCOR*, *PITX3*, and *PAX6*. Among them, eight were novel variants, and two were *de novo*. We confirmed the pathogenicity of *PAX6* intronic mutation using *in vitro* splice assays and redefined the pathogenicity of the variants of uncertain significance as likely pathogenic. Patients with developmental eye diseases typically have complex ocular and systemic phenotypes, possibly leading to significant visual impairment and high blindness rates. Identifying the genetic causes facilitates early diagnosis and effective treatment and rehabilitation strategies, thereby providing valuable insights for genetic and clinical counseling of these patients.

## 1. Introduction

Developmental eye disorders encompass structural defects of the eye, including the cornea, lens, iris, and optic fissure, which manifest at birth and are caused by abnormalities in the normal eye development process [1]. The spectrum of developmental eye disorders [1–3] includes microphthalmia, anophthalmia, and ocular coloboma (MAC); anterior segment dysgenesis (ASD) consisting of primary congenital glaucoma (PCG), aniridia, iridogoniodysgenesis, and Axenfeld–Rieger anomaly; congenital cataracts associated with microcornea; Leber congenital amaurosis (LCA); vitreoretinal dysplasia; optic nerve disorders; and infantile nystagmus, etc.

Developmental eye disorders can be unilateral or bilateral and are frequently responsible for severe visual impairment. Over 200 genes associated with non‐retinal developmental eye disorders have been reported [1, 3]. A previous study reported that the birth prevalence of MAC, the most severe form of developmental eye disorder, was 11.9 per 100,000 live births [4], with genes implicated in MAC including *SOX2*, *OTX2*, *BMP4*, *BMP7*, and *STRA6*, etc. PCG affects approximately 1 in 20,000 children [5], whereas congenital cataracts affect 3 in 10,000 children under 15 years in the United Kingdom [6]. Although these developmental disorders are rare, they collectively account for a significant proportion of global blindness. Despite considerable advances in the genetic diagnosis of retina‐associated developmental eye disorders by Chinese ophthalmologists, the genetic underpinnings of non‐retinal developmental eye disorders in Chinese patients remain underreported. Molecular diagnosis of non‐retinal developmental eye disorders in this population is particularly challenging because of a) limited access to medical advice and genetic testing due to financial constraints and b) diagnosis complexity arising from highly heterogeneous clinical presentations and diverse genetic causes, with many developmental eye disorders occurring as part of syndromes involving metabolic, developmental, physical, or sensory abnormalities [3].

In our previous study, we focused on the genetic diagnosis of inherited retinal diseases, confirming that next‐generation sequencing (NGS) technology combined with whole exome sequencing (WES) or a specific hereditary eye disease enrichment panel (HEDEP) can be employed to efficiently detect the genetic causes of inherited retinal dystrophies (IRDs) [7–10]. In this study, we aimed at reporting the clinical and genetic findings in a cohort of 14 Chinese families with non‐retinal developmental eye disorders. The patients exhibited complex ocular phenotypes with or without systemic manifestations. Among the cases, four probands were diagnosed with microphthalmia (with or without microcornea, congenital cataract, or congenital corneal opacification (CCO)). These phenotypes were caused by mutations in different genes. Additionally, we described six patients with ASD, including two probands with variants in the *PITX3* gene, one affected with Peter′s anomaly (PA) and the other with PA and microphthalmia: two patients with congenital iridocoloboma or aniridia (with microcornea and/or congenital cataract) and two patients with PCG. We also reported three patients with congenital cataracts (with CCO or microcorneas). Additionally, we described one patient with Rubinstein–Taybi syndrome (RSTS), caused by a variant in the *CREBBP* gene. Following the American College of Medical Genetics and Genomics and the Association for Molecular Pathology (ACMG/AMP) variant interpretation guidelines, we reported 17 (likely) pathogenic variants and one variant of uncertain significance (VUS) in 16 different genes. Among them, eight were reported for the first time in this study, and two were *de novo*. We also confirmed the pathogenicity of *PAX6* intronic mutation using an *in vitro* splice assay and redefined the pathogenicity of the variants of uncertain significance as likely pathogenic.

## 2. Materials and Methods

### 2.1. Ethics Statement

This study adhered to the principles of the Declaration of Helsinki. All experiments involving patient DNA and the DNA of their relatives were approved by the Peking University Third Hospital Medical Ethics Committee (No. 2012093). Written informed consent was obtained from all participants or from guardians in the case of minors. The ethics committee approved this consent procedure.

### 2.2. Patients

We recruited 14 probands from unrelated Chinese families of Han ethnicity affected by non‐retinal developmental eye disorders from the Department of Ophthalmology, Peking University Third Hospital.

We obtained detailed medical and family histories from probands or their family members. All patients underwent standard ophthalmic examinations, including best‐corrected visual acuity (BCVA), slit‐lamp biomicroscopy, intraocular pressure (IOP) measurement, dilated indirect ophthalmoscopy, and fundus photography, when possible. Additionally, patients underwent a systematic physical examination before genetic testing. We classified inheritance patterns based on the criteria described by Stone et al. [11] with minor modifications: (i) autosomal dominant (AD): a minimum of three generations with at least one instance of male‐to‐male transmission; (ii) autosomal recessive (AR): multiple affected patients within a single sibship with healthy parents; (iii) X‐linked (XL): affected males in multiple sibships connected through unaffected or mildly affected females, with no male‐to‐male transmission; and (iv) uncertain inheritance: sporadic cases or other multiplex kindreds with unclear inheritance patterns.

### 2.3. Molecular Genetics Analysis

We collected blood samples from all probands and available family members and extracted genomic DNA (gDNA) using standard protocols (D2492, Omega Bio‐Tek). A specific HEDEP based on exon capture technology was used to collect the protein coding regions of targeted genes. This HEDEP was able to capture 483 hereditary eye disease genes. We subjected the probands to HEDEP for targeted exon enrichment analysis or WES, which was performed using the Agilent SureSelect Human All Exon V6 kit (Agilent, United States) [8]. We performed NGS using an Illumina HiSeq X platform (Illumina, San Diego, California).

### 2.4. High Throughput Data Analysis and Variant Classification

We analyzed HEDEP sequencing data as previously described [8] and classified sequence changes according to the ACMG/AMP guidelines. We used PolyPhen‐2, SIFT, and MutationTaster for functional predictions.

### 2.5. Sanger Sequencing

We validated all variants considered likely to be disease‐causing using Sanger sequencing. Additionally, we used Sanger sequencing to determine whether the variant cosegregated with the disease phenotype in the available family members.

### 2.6. Conservation Analysis and Protein Modeling

We determined cross‐species conservation analyses of amino acids at the altered sites in different species using ConSurf (https://consurf.tau.ac.il/consurf_index.php). We predicted the 3D protein structures for the missense variant c.121G>A p. Gly41Ser in *CRYGC* and c.584G>A (p.R195H) in *EPHA2*, using Missense3D (http://missense3d.bc.ic.ac.uk/%7Emissense3d/). Additionally, we constructed 3D protein models of missense variants using AlphaFold3 (https://golgi.sandbox.google.com/) and predicted protein stability using DUET (http://biosig.unimelb.edu.au/duet/stability), DynaMut2 (https://biosig.lab.uq.edu.au/dynamut2/submit_prediction_mm), I‐Mutant2.0 (https://folding.biofold.org/i-mutant/i-mutant2.0.html), SAAFEC‐SEQ (http://compbio.clemson.edu/SAAFEC-SEQ/#started), and MUpro (https://mupro.proteomics.ics.uci.edu/).

### 2.7. Overexpression Vector Construction

For the *CRYGC* or *EPHA2* overexpression experiments, the full‐length *CRYGC* or *EPHA2* cDNA with or without variant was cloned into pEGFPN1 vectors using the pEASY‐Basic Seamless Cloning and Assembly Kit (CU‐201, TransGen Biotech, Beijing, China) according to the manufacturer′s protocol. The constructs were used to transform Trans1‐T1 phage‐resistant chemically competent cells (CD501; TransGen Biotech, Beijing, China).

### 2.8. Immunofluorescence (IF) Study

The HEK293A (293A) cells were transfected with a wild‐type (WT)/mutant *EPHA2*‐pEGFPN1 or *CRYGC*‐pEGFPN1 plasmids using PEI. After 24 h of transfection, the cells were fixed in 100% methanol (chilled at −20°C) at room temperature (RT) for 5 min, and the samples were incubated for 10 min with PBS containing 0.1% Triton X‐100. Cells were incubated with 5% normal donkey serum (017‐000‐121, Jackson Laboratory, United States) in PBST for 30 min at RT to block non‐specific binding of the antibodies. The primary antibodies used were monoclonal mouse anti‐p62 antibody (1:250 dilution, ab109012, Abcam, Cambridge, Massachusetts) and rabbit monoclonal anticalreticulin antibody (1:500 dilution, ab92516, Abcam, Cambridge, Massachusetts). The secondary antibodies used were donkey antimouse antibody conjugated with Alexa Fluor 568 (1:800 dilution, A10037, Invitrogen) and donkey antirabbit antibody conjugated with Alexa Fluor 568 (1:800 dilution, A10042, Invitrogen). Transfected cells were counterstained with DAPI (1:5000 dilution, C0060, Solarbio, Beijing, China) and examined under a Leica STELLARIS 5 confocal microscope (Leica, Germany).

### 2.9. WB Analysis

The 293A and HeLa cells were homogenized in 100 *μ*L of RIPA buffer (C1053+, Applygen, Beijing, China) plus protease inhibitors. Protein quantification was performed using a bicinchoninic acid (BCA) kit (P1513, Applygen, Beijing, China). Ten microgram of protein extract was loaded onto a 10% sodium dodecyl sulfate‐polyacrylamide gel. Following electrophoresis, proteins were transferred onto a PVDF membrane. The membrane was blocked with 5% milk in TBST (B1009, Applygen, Beijing, China) and incubated with a primary mouse monoclonal gamma C crystallin antibody (1:4000 dilution, MG135801, Abmart, China), mouse anti‐EphA2 (C3) antibody (1:1000 dilution, sc‐398832, Santa Cruz, California, United States), and mouse monoclonal anti‐GAPDH antibody (1:5000 dilution, ZL9002, ProteinTech Group, Chicago, United States) in primary antibody diluent for western (C1240, Applygen, Beijing, China) overnight at 4°C. The next day, after washing three times with 1 × TBST (B1109, Applygen, Beijing, China), the membrane was incubated with horse radish peroxidase (HRP)–conjugated goat antimouse IgG antibody (A0216, Beyotime, Shanghai, China) or goat antirabbit IgG antibody (A0208, Beyotime, Shanghai, China) at 1:1000 dilution for 1 h at RT. GAPDH was used as a loading control. Blots were analyzed using a ChemiDoc MP imaging system (Tanon Science & Technology Co., Shanghai, China).

### 2.10. *In Vitro* Splice Assay

As described previously [7], in an RNA splicing assay, we generated and combined a shortened *PAX6* intron DNA sequence around the splice acceptor site (Intron A, 200 bp) with another shortened intron DNA sequence around the splice donor site (Intron B, 200 bp). We combined Introns A and B to generate the shortened *PAX6* intron. We linearized the plasmid pmCherryN1 using an SbfI restriction enzyme (R3733S, New England Biolabs), followed by purification with a gel extraction kit (D2500, Omega). We cloned the shortened *PAX6* intron, with or without the mutation, into the SbfI site using a pEASY‐Basic Seamless Cloning and Assembly Kit (CU201, TransGen Biotech). We transfected 293T cells with the recombinant vectors using PEI (B600070; ProteinTech Group, Chicago, Illinois, United States). After 24 or 72 h of transfection, we observed mCherry expression under a fluorescence microscope (Nikon).

### 2.11. Minigene Construction

To further validate the effect of the *PAX6* intron variant, fragments of the upstream and downstream exons and introns containing the variant‐located introns were cloned, with WT fragments serving as templates for variant amplification. PCR was performed using KOD DNA polymerase (KFX‐101, TYOBO, Japan) according to the manufacturer′s protocol. Plasmid PX601 served as the plasmid backbone and was digested using two restriction enzymes, EcoRI and MfeI (New England Biolabs, Ipswich, Massachusetts, United States). The WT or mutant fragment amplicons were inserted into the digested PX601 using the Basic Seamless Cloning and Assembly Kit (CU201, TransGen Biotech, Beijing, China) according to the manufacturer′s protocol.

To investigate the splicing effect of the missense variant in *EPHA2* Exon 3, we amplified regions of Exon 2, Exon 3, Intron 4, and the 50 intronic nucleotides upstream and downstream of the canonical donor/acceptor splice sites with/without variant. Plasmid pSPL3 served as the plasmid backbone and was digested using two restriction enzymes, XhoI and BamHI (New England Biolabs, Ipswich, Massachusetts, USA). The WT or mutant fragment amplicons were inserted into the digested pSPL3 plasmid. The pSPL3 vector contains a small artificial gene composed of an SV40 promoter, an exon–intron–exon sequence with functional splice donor and acceptor sites, and a late polyadenylation signal. The single intron includes a multiple cloning site that can accommodate a genomic fragment to create a minigene expression construct.

### 2.12. Splicing Analysis

WT and mutant PX601‐minigene or pSPL3‐minigene plasmids were transfected into 293 cells at 80% confluency, using a concentration of 1 *μ*g/well in six‐well plates. Forty‐eight hours posttransfection, cells were collected. RNA was extracted and reverse‐transcribed into cDNA using the PrimeScript RT Reagent Kit (RR047A, Takara Bio Inc., Japan) following the manufacturer′s instructions. RT‐PCR was performed to determine *PAX6* or *EPHA2* cDNA isoforms. The transcripts were visualized on a 1% agarose gel.

### 2.13. Statistical Analysis

We evaluated the data using a two‐tailed unpaired Student′s *t*‐test, presented as the mean ± SEM. We used GraphPad Prism 9 (GraphPad Software, La Jolla, California, United States) for the statistical analyses, with statistical significance set at *p* < 0.05.

## 3. Results

### 3.1. Clinical Phenotypes of Chinese Patients With Non‐retinal Developmental Eye Disorders

In this cohort, we recruited six male and eight female probands from 14 unrelated Chinese families with non‐retinal developmental eye disorders. All patients had complex, overlapping ocular manifestations. Table S1 summarized the primary clinical information of the cohort. Among the patients, four were diagnosed with microphthalmia with or without microcornea and congenital cataract or CCO; six patients with ASD, including two probands affected with PA (with or without microphthalmia); two with congenital iridocoloboma or aniridia (with microcornea and/or congenital cataract); and two with PCG. Additionally, we reported three patients with congenital cataracts (with CCO or microcorneas) and one with RSTS. All probands suffered from developmental eye disorders at birth or during childhood. Most patients underwent ophthalmologic operations; however, they and/or their guardians complained of poor visual acuity. According to their clinical data, the inheritance patterns for 1, 5, and 8 cases were initially classified as AR, AD, and sporadic, respectively (Table S1, Figures 1 and 2).

**Figure 1:**
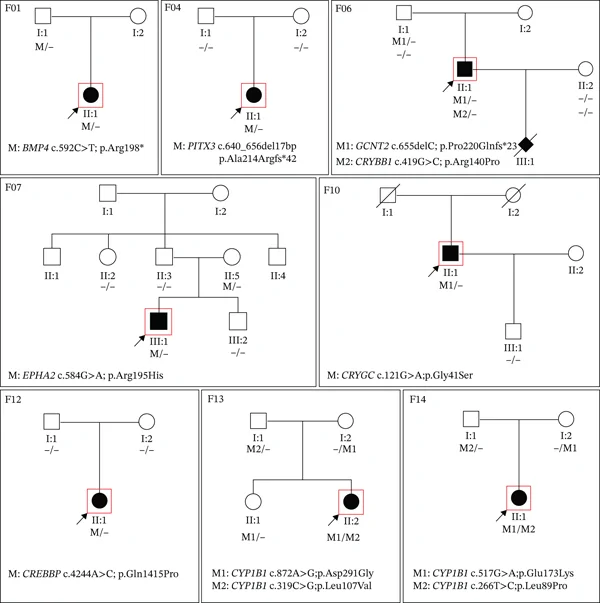
Pedigrees, genotypes, and segregation analysis for all sporadic developmental eye disorders families. Affected individuals are given with filled symbols, unaffected individuals are represented with open symbols, and individuals with a milder phenotype are given with gray symbols. Arrows indicate the proband. DNA numbers and genotypes for individuals with available DNA are given below each symbol.

**Figure 2:**
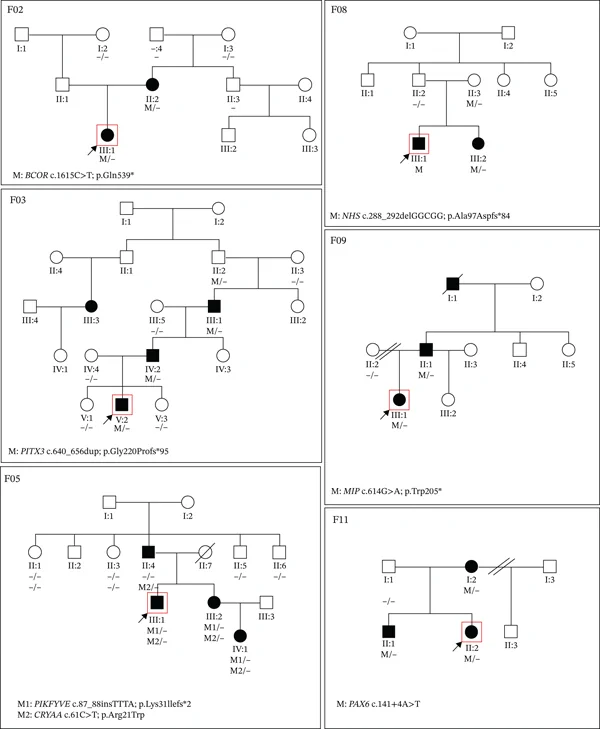
Pedigrees, genotypes, and segregation analysis for AR and AD developmental eye disorders families. Affected individuals are given with filled symbols; unaffected individuals are represented with open symbols and individuals with a milder phenotype are given with gray symbols. Arrows indicate the proband. DNA numbers and genotypes for individuals with available DNA are given below each symbol. AR, autosomal recessive; AD, autosomal dominant.

### 3.2. Molecular Diagnosis of Chinese Patients With Non‐retinal Developmental Eye Disorders

Among the eight sporadic cases, we identified six probands with heterozygous mutations in *BMP4* (F01), *PITX3* (F04), *CRYBB1* and *GCNT2* simultaneously (F06), *EPHA2* (F07), *CRYGC* (F10), and *CREBBP* (F12). Based on this finding, their inheritance patterns were redefined as AD. We observed that two probands (F13 and F14) with PCG harbored compound heterozygous variants in *CYP1B1,* therefore, their inheritance patterns were redefined as AR (Table S1, Figure 1). Five families whose inheritance modes were initially defined as AD were determined to harbor (likely) disease‐causing variants in *BCOR* (F02), *PITX3* (F03), *CRYAA* and *PIKFYVE* simultaneously (F05), *MIP* (F09, Figure S1), and *PAX6* (F11) genes (Table S1, Figure 2). We redefined their inheritance patterns as XL for F02 and AD for the others. The proband of family F08, a 33‐year‐old male with microphthalmia, congenital cataract, and CCO, was found to have a disease‐causing hemizygous variant in the *NHS* gene (Figure 2, F08; Figure S2). His parents were unaffected, but his younger sister had a similar condition. Genetic testing revealed that the variant in *NHS* was inherited from their mother (Figure 2, F08). The mother, a 65‐year‐old female, did not exhibit microphthalmia or microcornea. Consequently, we redefined the AR inheritance pattern for family F08 as XL.

Our study identified nine missense variants, five frameshift variants, three nonsense variants, and one intronic variant in 16 different genes (Table S1, Figure 1, and Figure 2). According to the ACMG/AMP guidelines, we defined 11 variants as likely pathogenic, six as pathogenic, and one as VUS (Tables S1 and S2). Eight variants were reported for the first time in this study (Table S1). Two probands (Figure 2, F05; Figure 1, F06) had likely pathogenic variants in two genes simultaneously. The proband of family F05 was affected by microphthalmia with microcornea and congenital cataracts, as well as nystagmus (Table S1; Figure S3A,B). His sister (III:2) and the sister′s daughter (IV:1) had similar clinical symptoms, whereas his father (II:4) was affected by bilateral congenital cataracts. Genetic testing showed that the proband, individuals III:2 and IV:1, had the disease‐causing variant c.61C>T (p. Arg21Trp) in *CRYAA* (Figure 2, F05; Figure S3C), which was inherited from individual II:4. The variant has been reported to cause congenital cataract and microphthalmia with microcornea [12, 13]. The proband underwent bilateral phacoemulsification combined with intraocular lens implantation and penetrating keratoplasty (PKP) in the left eye following trauma. Despite carrying a likely pathogenic variant c.87_88insTTTA (p. Lys31Ilefs∗2) in *PIKFYVE*, clinical examinations of the proband and individuals III:2 and IV:1 did not indicate corneal dystrophy (Figure S3A,B), although they all carried a likely pathogenic variant (Figure 2, F05; Figure S3C). The proband of family F06 was affected by congenital cataracts and microcornea (Figure S4A). Genetic testing identified two disease‐causing variants: c.655delC (p. Pro220Glnfs∗23) in *GCNT2* inherited from his father and c.419G>C (p. Arg140Pro) in *CRYBB1* (Figure 1, F06; Figure S4B). The variant p. Arg140Pro in *CRYBB1* was novel, with multiple orthologous sequence alignments demonstrating that this position was highly conserved among all species (Figure S4C). The proband′s father did not report congenital cataracts. The proband underwent bilateral phacoemulsification combined with intraocular lens implantation and pars plana vitrectomy (PPV) with silicone oil in the right eye due to retinal detachment.

### 3.3. Several Illustrative Cases

#### 3.3.1. Family F01 and Family F02

The proband of family F01 was a 4‐year‐old female diagnosed with microphthalmia with microcornea and CCO (Figure 3A,B). Bilateral axial length (AL) taken at the age of 2 years was less than 15 mm. Her parents reported growth and developmental delays, including intellectual development. She underwent PKP in her right eye when she was 4 years old, and as the disease progressed, she developed a right posterior scleral staphyloma. Genetic testing revealed a heterozygous variant c.592C>T (p. Arg198∗) in *BMP4*, inherited from her father (Figure 1, F01; Figure 3C). Although the father had myopia, he did not present with developmental eye disorders. This variant has been previously reported in cleft lip/palate [14], anophthalmia/severe microphthalmia, mild microphthalmia with sclerocornea, facial asymmetry, and right‐sided diaphragmatic hernia [15]. The proband of family F02 was a 3‐year‐old female with a diagnosis of microphthalmia, congenital cataract and microcornea, and nystagmus. Genetic testing identified a heterozygous variant c.1615C>T (p. Gln539∗) in *BCOR* (Figure 2, F02; Figure 3D), which was classified as pathogenic based on ACMG/AMP guidelines. This variant alters a conserved amino acid (Figure 3E) and was first reported in this study. The proband′s mother also had the same variant and was affected by congenital cataracts.

**Figure 3:**
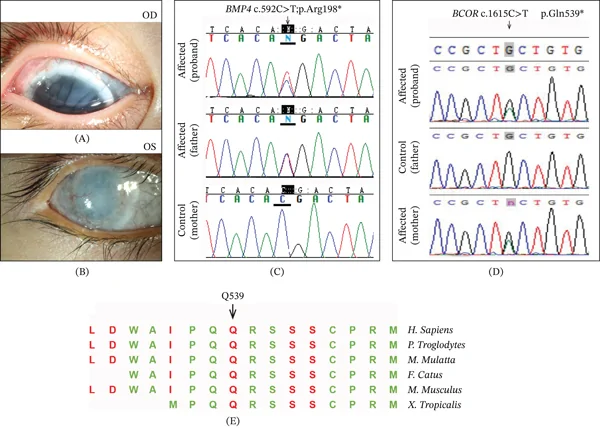
The clinical and genetic findings of the proband in family F01 and F02. Anterior segment photography of the proband in family F01 showed the patient underwent PKP in her (A) right eye and CCO in her (B) left eye. (C) Sanger sequencing showed the proband in family F01 had a heterozygous variant in *BMP4*, inherited from her father, and (D) the proband in family F02 had a heterozygous variant in *BCOR*, inherited from her mother. (E) Multiple orthologous sequence alignment of *BCOR* around the Q539 amino acid in several species.

#### 3.3.2. Family F03 and Family F04

The proband of family F03, a 3‐year male, was affected by PA (Figure 4A,B,C, and D) and microphthalmia. His bilateral AL was less than 18 mm when he was 1 year old. Genetic testing showed that he harbored a heterozygous variant c.640_656dup (p. Gly220Profs∗95) in *PITX3* (Figure 2, F03; Figure 4 E), inherited from his father. Several individuals in this family presented with similar symptoms and carried the same variant. This variant was previously reported in patients with ASD, including PA and congenital cataracts [16, 17]. In family F04, the proband was a 3‐year‐old female with PA (Figure 4F,G,H, and I). Genetic testing revealed a *de novo* heterozygous variant c.640_656del17bp (p. Ala214Argfs∗42) in *PITX3* (Figure 1, F04; Figure 4J).

**Figure 4:**
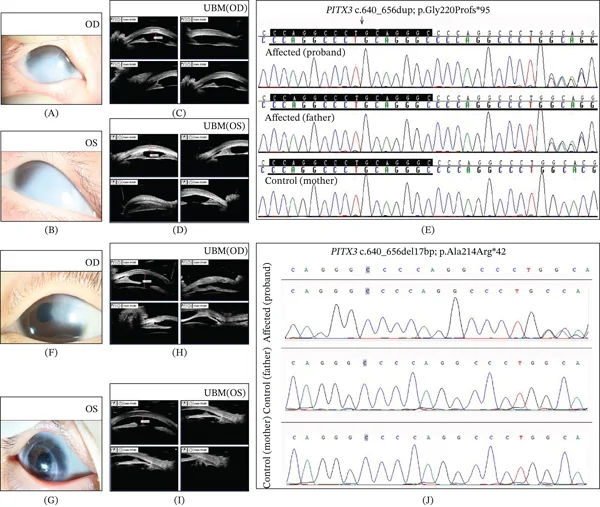
The clinical and genetic findings of the proband in family F03 and F04. Anterior segment photography and UBM result of the proband in family F03 showed the patient had bilateral PA including CCO (A‐D) and anterior synechia of iris (C‐D); Sanger sequencing showed the proband in family F03 had a heterozygous variant in *PITX3*, inherited from his father (E); Anterior segment photography and UBM of the proband in family F04 showed the patient had bilateral PA including CCO (F‐I) and anterior synechia of iris (H‐I) and underwent PKP in her left eye (G); (J) Sanger sequencing showed the proband in family F04 had a de novo heterozygous variant in *PITX3*.

#### 3.3.3. Family F11

The proband of family F11 was a 33‐year‐old female diagnosed with aniridia, microcornea, and congenital cataract (Figure 5A). Genetic testing showed that the patient and her affected older brother had a heterozygous variant c.141+4A>T in *PAX6*, inherited from their mother (Figure 2, F11; Figure 5B). The intronic variant disrupts the splice donor site of Exon 5 in *PAX6*, thereby becoming pathogenic in patients with hereditary aniridia [18, 19]. In this study, we further investigated the intronic pathogenic variant using an *in vitro* splicing assay. We cloned WT or mutant variant of a shortened version of Intron 5 into the mCherry coding sequence of a reporter construct (Figure 5C). Sanger sequencing results of reporter plasmids with the intronic variant are shown in Figure 5D. If not successfully spliced, the presence of the intron would result in a premature stop codon and therefore would not generate mCherry expression. Successful splicing would enable complete removal of the intron and lead to mCherry expression. As illustrated in Figure 5E, we observed mCherry expression in 293T cells transfected with WT‐intron‐pmCherryN1 but not in 293T cells transfected with mutant‐intron‐pmCherryN1. We observed that 72 h after transfection, the number of mCherry+ cells per random sight in 293T cells transfected with mutant‐intron‐pmCherryN1 plasmids was significantly lower than that in 293T cells transfected with the WT‐intron‐pmCherryN1 plasmid (Figure 5F), indicating that the intronic variant disrupted *PAX6* splicing effect. In addition, minigene assay (Figure 5G) result indicated that the variant c.141+4A>T led to two abnormal products (Figure 5H) including a pseudoexon (PE) inclusion of 42 bp, which was part of Intron 5 (P1), causing a frameshift insertion and Exon 5 skipping (P2), causing an in‐frame deletion of 131 bp (Figure 5I).

Figure 5Clinical and genetic findings in family F11. (A) Anterior segment photography of the proband showed the patient had aniridia and congenital cataract. (B) Sanger sequencing showed the proband had a heterozygous intronic variant in *PAX6*. (C) Schematic view of pmCherryN1 plasmid used in the splicing assay. A shortened *PAX6* intron was generated for use in a splicing assay by selecting DNA sequence around the splice acceptor site (Intron A), which was combined with DNA sequence around the splice donor site (Intron B). (D) Sanger sequencing indicated that Intron 5A contained the c.141+4A>T variant (lower). (E) The mCherry expression in 293T cells transfected with pmCherryN1 vector containing WT or mutant intron, 24, 48 and 72 h after transfection. (F) The number of mCherry+ cells per random sight in 293T cells transfected with mutant plasmid was statistically lower than that in 293T cells with WT plasmid. Error bars show SEM, and the significance was calculated using two‐tailed unpaired *t*‐test,  ^∗∗∗∗^ *p* < 0.001. (G) Schematic view of PX601‐*PAX6* minigene plasmid. (H,I) RT‐PCR analysis for the variant which was predicted to affect splicing. “P1” and “P2” as different splicing products.

**Figure:**
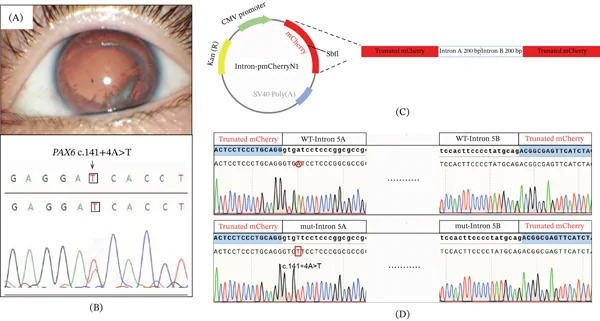

**Figure:**
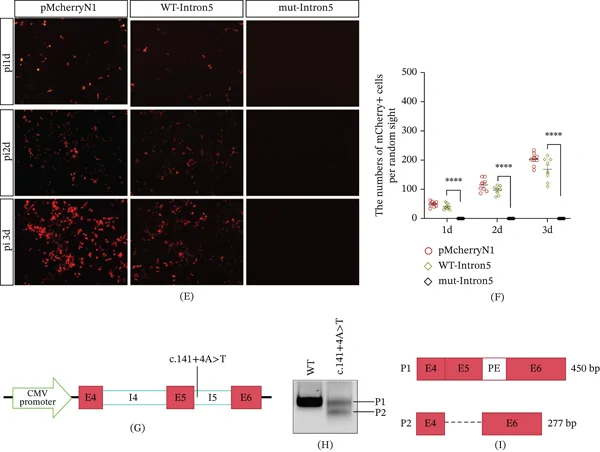

#### 3.3.4. Family F12

The proband of family F12 was a 7‐year‐old female with complex clinical manifestations including CCO, congenital glaucoma, optic disc dysplasia, congenital vitreous anomalies, developmental delay, language and motor ability development delay, cognitive impairments, EEG abnormalities, and autism. However, the exact clinical diagnosis remained unclear. She was born at 38 weeks of gestation with a weight of 2.9 kg and a length of 50 cm. At birth, the parents noticed she had CCO in the left eye. At 2 months of age, she was diagnosed with familial exudative vitreoretinopathy (FEVR) and underwent bilateral intravitreal injections (ranibizumab). At the age of 9 months, her bilateral corneal diameters were 13 mm, with IOP of the right and left eyes being approximately 14 and 11 mmHg, respectively. Ultrasound biomicroscopy (UBM) revealed CCO and anterior synechia of the iris in her left eye (Figure 6A), with a left corneal diameter of approximately 11 mm. Fundus photo examination showed optic disc dysplasia in her right eye (Figure 6B). B‐ultrasound examination revealed bilateral congenital vitreous abnormalities (Figure S5A), and she subsequently underwent PKP due to CCO. The patient was diagnosed with congenital glaucoma and underwent trabeculotomy and Schlemm′s tuboplasty at the ages of 4 and 5 years in her left and right eyes, respectively. Her right corneal diameter was 14 mm and 15 mm at ages 4 and 5 years, respectively (Figure 6C), with IOP readings of approximately 35 mmHg and 31 mmHg in the right and left eyes, respectively. As the disease progressed, the patient developed bilateral progressive posterior scleral staphyloma (Figure S5B,C). At the age of 7 years, she underwent PPV with silicone oil and lens cutting in the right eye owing to endophthalmitis (Figure 6C). In addition to the complex ocular phenotypes, the patient had systemic phenotypes, as listed above. She also had broad thumbs and persistent fetal finger pads (Figure 6D). Genetic testing results indicated that she harbored a pathogenic heterozygous variant c.4244A>C (p. Gln1415Pro) in *CREBBP* (Figure 6E), and her parents were unaffected (Figure 1, F12). The variant has been reported to cause RSTS [20]. Combining the proband′s complex clinical symptoms, she was ultimately diagnosed with RSTS.

**Figure 6:**
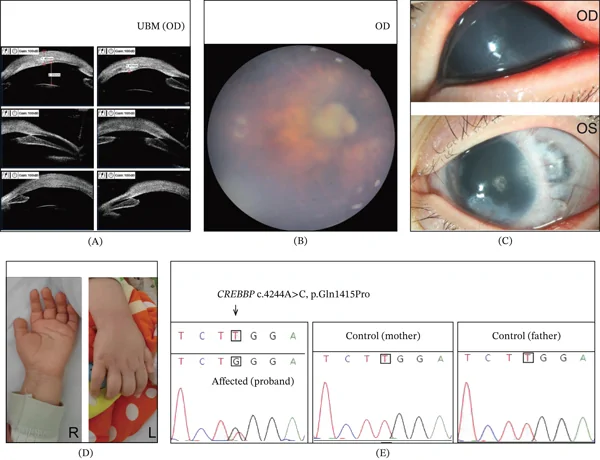
The clinical and genetic findings of the proband in family F12. The UBM and fundus photograph result of the proband showed the patient had CCO and anterior synechia of iris (A) and optic disc dysplasia (B) in her right eye, at the age of eight months; (C) Anterior segment photography showed the proband had bilateral increased corneal diameter and underwent PKP in her left eye; (D) The girl had broad thumbs, persistent fetal fnger pads; (E) Sanger sequencing showed the proband had a de novo heterozygous variant in *CREBBP*.

#### 3.3.5. Family F13 and F14

The probands of families F13 and F14 were affected by PCG (Table S1). In family F13, the proband was a 6‐month‐old female with CCO in the right eye and bilateral increases in corneal diameter since birth (Figure 7A). UBM revealed corneal edema and congenital developmental abnormalities in the anterior chamber angle of the right eye (Figure 7B). B‐ultrasound examination showed no obvious abnormalities in the posterior segment (Figure 7C). Bilateral ALs were measured as 20.73 mm (right) and 20.96 mm (left) at 6 months of age (Figure 7C). The IOP in the right and left eyes were 22 and 23 mmHg, respectively. Genetic testing revealed compound heterozygous variants c.872A>G (p. Asp291Gly) and c.319C>G (p. Leu107Val) in *CYP1B1* inherited from her mother and father, respectively (Figure 1, F13 and Figure 7D). According to the ACMG/AMP guidelines, these two variants were classified as pathogenic and VUS, respectively. The variant Leu107Val has been previously reported in patients with PCG [21, 22]; one study classified it as VUS. Functional experiments should be employed to validate its pathogenicity. The proband of family F14, a 4‐year‐old female, was also diagnosed as PCG, with genetic testing revealing pathogenic compound heterozygous variants c.517G>A (p. Glu173Lys) [23, 24] and c.266T>C (p. Leu89Pro) [25, 26] (Figure 1, F14; Figure 7E) in *CYP1B1*.

**Figure 7:**
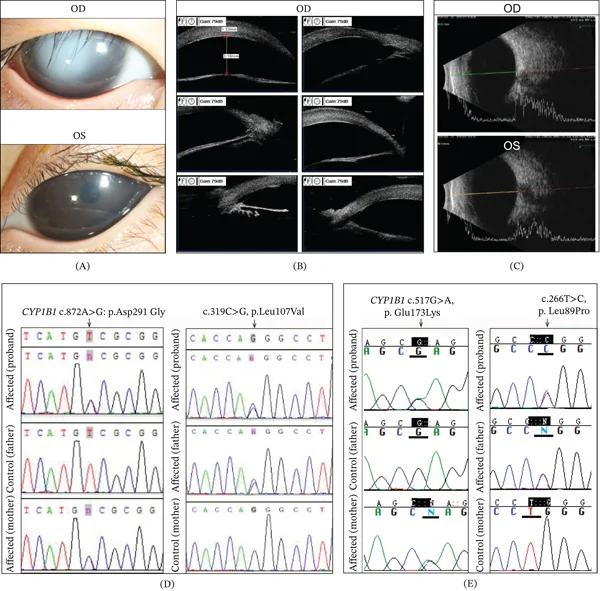
The clinical and genetic findings of the proband in family F13 and F14. (A) Anterior segment photography showed that the proband in family F13 had CCO in the right eye and bilateral increases corneal diameter; (B) The UBM examination result revealed corneal edema, congenital developmental abnormalities of the anterior chamber angle in the right eye of the proband in family F13; (C) The B‐ultrasound examination result of the proband in family F13; Sanger sequencing showed the proband in family F13 (D) and F14 (E) had compound heterozygous variants in *CYP1B1*.

### 3.4. Redefine the Pathogenicity of VUS in Two Cases

#### 3.4.1. Family F07

The proband of family F07 was a 31‐year‐old male diagnosed with congenital cataract and microcornea (Figure 8A). He underwent lens extraction surgery at the age of 1 year. Genetic testing revealed a heterozygous variant c.584G>A (p. Arg195His) in *EPHA2* inherited from his asymptomatic mother (Figure 1, F07 and Figure 8B). This variant was first reported in this study, and multiple orthologous sequence alignments demonstrated that this position was highly conserved (Figure 8C). The variant yielded a high pathogenicity score using the online SIFT, PolyPhen‐2, and MutationTaster prediction programs. Arg195 is located in the cd10480 domain of EPHA2 protein. 3D structural modeling of EPHA2 using the AlphaFold3 database revealed that the hydrogen bond between the main chain His195 and Glu28 in the mutant protein was broken (Figure 8D), indicating the variant would affect the main chain. Additionally, the surface electrostatic potential of the 195th amino acid in the WT protein was electronegative, whereas the mutation increased the electronegativity at these sites (Figure 8E), suggesting that the mutation affected the surface electrostatic potential of the EPHA2 protein. The influence of the variant Arg195His on the stability of EPHA2 protein was analyzed using DUET, MUpro, DynaMut2, and I‐Mutant2.0 SEQ databases. A negative value indicated that the protein stability decreased, suggesting that the mutation affected the protein stability (Figure 8F). According to the ACMG/AMP guidelines, the variant is currently classified as VUS (Table S2). The pSPL3 minigene assay result indicated that the missense variant showed no abnormal product in this study (Figure 8G,H). Localization study verified that both GFP‐tagged WT and mutant EPHA2 proteins were distributed across the plasma membrane and cytoplasm in 293A and HeLa cells. However, the two cell lines transfected with the mutant plasmid exhibited diminished cytoplasmic GFP fluorescence intensity, thereby indicative of the reduced intracellular protein accumulation of the mutant EPHA2 protein (Figure 8I,J). This observation was consistent with WB result, which demonstrated a mutant protein reduction, when compared with its WT counterpart (Figure 8K). Above all, we confirmed the decreased stability of the mutant protein using in vitro experiments. According to ACMG guideline, we classified this evidence as PS3, and ultimately, the mutation was reclassified as likely pathogenic variant (Table S2).

**Figure 8:**
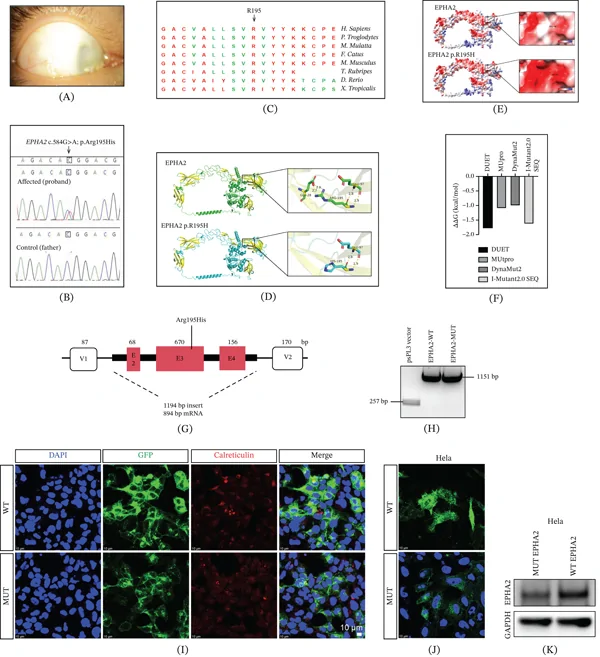
The clinical and genetic findings of the proband in family F07. (A) Anterior segment photography showed that the proband in family F07 had microcornea and strabismus in the left eye; (B) Sanger sequencing showed the proband in family F07 had a heterozygous variant in *EPHA2*; (C) Multiple orthologous sequence alignment of *EPHA2* around the R195 amino acid in several species; (D) The 3‐D structure of *EPHA2* protein; (E) The surface electrostatic potential of the 195th amino acid in WT and mutant *EPHA2* protein, blue shows areas of positive charge distribution, red shows areas of negative charge distribution, and white shows areas with no charge; (F) The influence of the variant Arg195His on the stability of *EPHA2* protein, the output result was expressed as ΔΔG(kcal/mol), and the negative value indicated that the protein stability decreased. (G) Schematic view of pSPL3‐*EPHA2* minigene plasmid. (H) RT‐PCR analysis for *EPHA2* variant, vector, *EPHA2*‐WT and ‐MUT indicated 293A cells transfected with pSPL3 vector, WT and mutant *EPHA2*‐ pSPL3 plasmids, respectively. IF staining result of 293A (I) and Hela (J) cells transfected with WT and mutant *EPHA2*‐pEGFPN1 plasmid, endoplasmic reticulum were labeled with Anti‐Calreticulin antibody (red), nuclei were stained blue by DAPI, Scale bar = 10 μm. WB result of Hela (K) cells transfected with WT and mutant *EPHA2*‐pEGFPN1 plasmids.

#### 3.4.2. Family F10

The proband of family F10, a 63‐year‐old male, was diagnosed with congenital cataract and microcornea, as well as iridocoloboma. Genetic testing revealed a heterozygous variant c.121G>A (p. Gly41Ser) in *CRYGC* (Figure 1, F10 and Figure 9A). The variant Gly41Ser was in the XTALbg domain of the CRYGC protein, and multiple orthologous sequence alignments demonstrated that this position was highly conserved (Figure 9B). 3D structural analysis indicated that the variant might not affect the main chain structure (Figure 9C) and that the mutation did not affect the surface electrostatic potential of the CRYGC protein (Figure 9D). The influence of the variant Gly41Ser on the stability of CRYGC protein was also analyzed, suggesting that the mutation affected protein stability (Figure 9E). The variant yielded a high pathogenicity score using the online SIFT, PolyPhen‐2, and MutationTaster prediction programs. According to the ACMG/AMP guidelines, the variant was identified as VUS (Table S2). The GFP‐tagged WT CRYGC protein displayed a consistent distribution pattern, being uniformly dispersed in both the cytosol and nucleus (Figure 9F); however, the mutant protein was prone to form intracellular aggregates, which were colocalized with p62 (the aggregate marker). Consistent with the phenomenon observed from confocal microscope snapshots, compared with the WT protein which mainly appeared in the supernatant, the expression of the mutant protein in the supernatant was significantly reduced, either in 293A (Figure 9G) and HeLa (Figure 9H) cells. According to ACMG guideline, we classified this evidence as PS3, and ultimately, the mutation was reclassified as likely pathogenic variant (Table S2).

**Figure 9:**
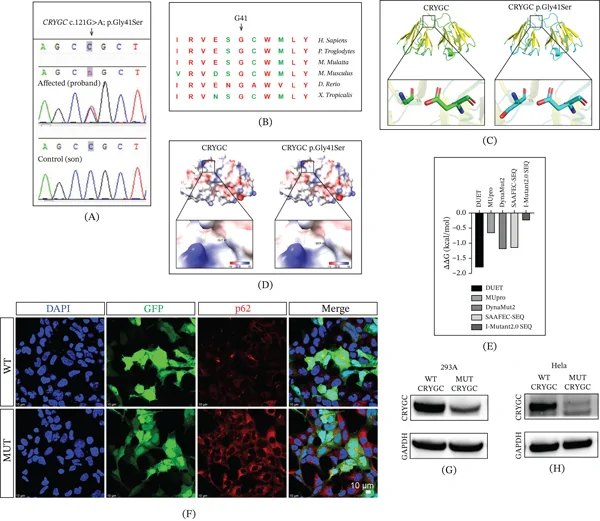
The clinical and genetic findings of the proband in family F10. (A) Sanger sequencing showed the proband in family F10 had a heterozygous variant in *CRYGC*. (B) Multiple orthologous sequence alignment of *CRYGC* around the G41 amino acid in several species. (C) The 3‐D structure of *CRYGC* protein. (D) the surface electrostatic potential of the 41th amino acid in WT and mutant *CRYGC* protein. (E) The influence of the variant Gly41Ser on the stability of *CRYGC* protein. (F) IF staining result of 293A cells expressing GFP‐tagged WT and mutant proteins. The aggregate was labeled with Anti‐p62 antibody (red), nuclei were stained blue by DAPI, Scale bar = 10 μm. WB result of 293A (G) and Hela (H) cells transfected with WT and mutant *CRYGC*‐pEGFPN1 plasmids.

## 4. Discussion

This study recruited 14 patients from different families who were diagnosed with developmental eye disorders, including microphthalmia, ASD, congenital cataracts, and RSTS, etc. These patients had many complex ocular phenotypes and poor vision since childhood, and although they underwent multiple ophthalmologic surgeries, they still suffered poor prognoses and even blindness. In this study, we identified 17 (likely) pathogenic variants and one VUS in 16 different genes, including *BMP4*, *BCOR*, *PITX3*, and *PAX6*. Among these, eight variants were newly identified in this study, and two were *de novo.* Additionally, we confirmed the pathogenicity of *PAX6* intronic mutations using an *in vitro* splice assay.

Our study reported four cases in which the proband was affected by microphthalmia and other associated anomalies caused by variants in different genes, with the exception of family F03 (Table S1). MAC is characterized by a smaller or absent eye or a missing portion of the eye structure that can be limited to one or involve multiple tissues [27]. Previous studies have reported that MAC conditions are often associated with additional ocular and nonocular anomalies, indicating the involvement of the responsible genes in multiple developmental processes [27]. Presently, more than 80 genes have been reported to cause MAC, with disruptions of developmental steps caused by human gene mutations in conserved genetic pathways predictably resulting in MAC [27, 28]. The *BMP4* gene encodes a secreted ligand of the transforming growth factor‐beta superfamily of proteins and regulates gene expression. Since *BMP4* has been shown to have various roles in embryonic and adult tissues, its involvement in multiple phenotypes, including orofacial cleft, microphthalmia, multiple cardiovascular diseases, and human cancers, is highly possible [15, 29–31]. The nonsense mutation c.592C>T (p. Arg198∗) in *BMP4* has previously been reported in a patient with cleft lip and palate [14], whereas the report by Reis et al. [15] showed that this mutation is associated with syndromic ocular phenotypes. Although our data support the association of the mutation Arg198∗ with non‐syndromic ocular phenotypes in the proband of family F01, a previous study indicated that some missense variants in *BMP4* represent rare polymorphisms rather than pathogenic mutations [15]. Therefore, some individuals with *BMP4* missense mutation can be unaffected. Other studies [15, 32] have demonstrated that frameshift mutations could lead to highly variable phenotypes; for instance, affected probands carrying *BMP4* frameshift mutations had unaffected parents with the same variant, indicating variable expressivity/incomplete penetrance of *BMP4* missense/frameshift mutations. In family F01, the father of the proband with the nonsense variant Arg198∗ was unaffected, which is the first report of a *BMP4* nonsense mutation in an apparently unaffected parent, supporting the variable expressivity/incomplete penetrance of *BMP4* nonsense mutation.

XL dominant oculofaciocardiodental (OFCD) syndrome caused by loss‐of‐function (including nonsense, splicing, frameshift, or deletions of part or all of the coding sequence) variants in the *BCOR* gene affects only females, with presumed embryonic lethality among males [14]. A previous study [12] reported that the heterozygous frameshift deletion c.4390_4393del (p.Glu1464Profs∗19) in *BCOR* resulted in nonsystemic ocular manifestations, including microphthalmia, cataracts, and glaucoma in a female. Another study [33] also reported a female affected with a severe ocular phenotype without systemic phenotypes caused by biallelic mosaic variants in *BCOR*. Additionally, male patients with XL recessive Lenz microphthalmia syndrome and male patients without microphthalmia caused by the specific missense variant c.254C>T (p. Pro85Leu) have been reported [34, 35]. These findings underscore the clinical and genetic heterogeneity of *BCOR*‐associated phenotypes. Our study reported for the first time a female proband in family F02 with a novel heterozygous nonsense variant in *BCOR* who suffered from nonsystemic microphthalmia, microcornea, and congenital cataracts. Her mother had a congenital cataract in the right eye, expanding the phenotypic spectrum of the *BCOR* gene. Our study also reported two probands (F05 and F08) with microphthalmia and congenital cataracts (with microcornea or CCO) caused by heterozygous variants in typical congenital cataract‐associated genes, including *CRYAA* and *NHS*. Previously, variants in these genes have been reported to result in microphthalmia in different races [36–41]. The variant c.61C>T (p.Arg21Trp) in *CRYAA* was reported to cause dominant congenital cataracts with/without microcornea [12, 13], whereas the variant c.288_292delGGCGG (p.Ala97Aspfs∗84) in *NHS* was novel. Although variants in *NHS* can cause Nance–Horan syndrome, the proband of family F08 exhibited complex ocular anomalies. Therefore, our findings expand the phenotypic and mutational spectra of these two genes.

In addition to the above patients with microphthalmia and associated anomalies, our study identified a proband affected by microphthalmia and PA simultaneously in an AD inheritance pattern. Genetic testing showed that the probands and affected individuals subjected to HEDEP had the heterozygous variant c.640_656dup (p. Gly220Profs∗95). This variant has been previously reported to cause dominant ASD, cataracts, and even PA [16, 17, 42]. *PITX3*, a paired‐like homeodomain transcription factor belonging to the RIEG/PITX homeobox gene family, is essential for normal embryonic eye and brain development in vertebrates [27]. The pathogenicity of *PITX3* heterozygous variants has been attributed to haploinsufficiency [16]. Patients with homozygous mutation c.650delG (p. Gly217Alafs∗92) in *PITX3* exhibited considerably more severe phenotypes than their heterozygous family members. These patients were affected with bilateral microphthalmia (along with corneal opacity) and severe developmental delay or neurological deficits, whereas their carrier parents were both affected with congenital cataracts [43]. Similarly, another homozygous mutation c.640_656del (p. Ala214Argfs∗42) in *PITX3* has been reported in an affected child with severe microphthalmia and sclerocornea, with her carrier parents (consanguineous marriage) being unaffected [13]. Our study is the first report of a heterozygous *PITX3* mutation c.640_656dup (p. Gly220Profs∗95) associated with dominant severe phenotypes including PA and microphthalmia (F03). We also reported a patient (F04) with the *de novo* heterozygous variant c.640_656del (p. Ala214Argfs∗42) in *PITX3* who was affected with PA. This variant has also been reported to cause dominant congenital cataracts in two separate Chinese families [44]. Our findings demonstrated that the variant also resulted in PA in a dominant pattern.

Furthermore, we reported a *de novo* pathogenic variant of *PITX3* and *CREBBP*. *De novo* variants, in other words, spontaneous variants, are now a well‐recognized cause of severe early‐onset genetic diseases [16]. These variants may be inherited from unaffected parents with germline mosaicism. Any genetic variation in the genomes of germinal cells within an individual is termed germline mosaicism. It is a driving force behind evolution and a fundamental cause of genetic diseases [17]. Approximately 80% of all *de novo* germline single nucleotide variants arise on the paternal allele, with advanced paternal age being a major factor linked to the increase in the number of de novo variants [45, 46]. Additionally, *de novo* mutations can also occur postzygotically during embryo development, throughout fetal development, and postnatally, extending through to death. These mutations are not evident in the parents or in the zygote of the affected individuals and are more precisely characterized as somatic mutations [17]. Previous studies have indicated that most RSTS cases are sporadic and are caused by de novo missense/nonsense mutations in *CREBBP* [47, 48]. Germline mosaicism is particularly difficult to evaluate, especially if a maternally originated mutation is suspected. This challenge results from the difficulty in obtaining eggs from ovaries, as well as the procedure being highly invasive. However, germline mosaicism should be suspected when two or more affected children are born to apparently unaffected parents [49]. In this study, we emphasize the significance of genetic testing in these families to identify the genetic causes.

Future studies should focus on expanding the genetic and phenotypic spectrum of developmental eye disorders by investigating additional rare variants and their roles in disease progression. Further research into the mechanisms of germline mosaicism and its impact on genetic inheritance, particularly in *de novo* mutations, could provide deeper insights into the variability and penetrance of these conditions. Additionally, exploring the functional consequences of newly identified mutations through advanced *in vitro* models and phenotypic assessments would help clarify their pathogenicity. Longitudinal studies involving diverse populations are also essential to better understand the environmental and genetic factors that contribute to disease variability and treatment outcomes. Finally, integrating genomic data with clinical findings could enhance personalized medicine approaches, improving diagnosis, treatment, and genetic counseling for affected individuals.

## Funding

This study was funded by the National Natural Science Foundation of China (82401215 to LXZ), the Peking University Medicine Sailing Program for Young Scholars′ Scientific and Technological Innovation, and the Fundamental Research Funds for the Central Universities (BMU2024YFJHPY019 to LXZ).

## Conflicts of Interest

The authors declare no conflicts of interest.

## Supporting Information

Additional supporting information can be found online in the Supporting Information section.

## Supporting information


**Supporting Information 1** Figure S1: Genetic testing results of family F09. Figure S2: Genetic testing results of family F08. Figure S3: Clinical and genetic testing results of family F05. Figure S4: Clinical and genetic testing results of family F06. Figure S5: B‐ultrasound examination results of the proband in family F12.

**Supporting Information 2** Table S1: Clinical information and genetic causes of 14 unrelated families with developmental eye diseases.

**Supporting Information 3** Table S2: The classification of variants according to ACMG and the AMP variant interpretation guidelines in this study.

## Data Availability

The data that support the findings of this study are available from the corresponding author on reasonable request.
