# Supplementary material for: Next‐Generation Sequencing to Identify Genetic Causes of Chinese Patients Affected With Non‐retinal Developmental Eye Disorders
Source: Hum Mutat. 2026 Oct 16;2026:4035027. doi: 10.1155/humu/4035027 (PMC13673294; doi:10.1155/humu/4035027)
Supplement: Supplementary file 1 [file HUMU-2026-4035027-s001.docx]

**Supplementary files**

**Fig.S1. Genetic testing results of family F09.** Sanger Sequencing showed that the proband in family F09 had a heterozygous variant in *MIP*, inherited from her father.

**Fig.S2. Genetic testing results of family F08.** Sanger Sequencing showed that the proband in family F08 had a hemizygous variant in *NHS*, inherited from his mother.

**Fig.S3. Clinical and genetic testing results of family F05.** (A-B) Anterior segment photography in the right and left eye of the proband in family F05 and he underwent PKP in the left eye. (C) Sanger Sequencing showed that the proband in family F05 had a heterozygous variant in *PIKFYVE* and *CRYAA*.

**Fig.S4. Clinical and genetic testing results of family F06.** (A) Anterior segment photography in the right eye of the proband in family F06; (B) Sanger Sequencing showed that the proband in family F06 had a heterozygous variant in *GCNT2* and *CRYBB1*. (C) Multiple orthologous sequence alignment of CRYBB1 around the R140 amino acid in several species.

**Fig.S5. B ultrasound examination results of the proband in family F12** at 8 months (A), 6 years and 11 months (B),7 years (C) of age.
